# Traumatic Obturator Hip Dislocation: Injury Characteristics and Clinical Outcomes

**DOI:** 10.7759/cureus.83852

**Published:** 2025-05-10

**Authors:** Vera Jaecker, Marcel Niemann, Bertil Bouillon, Sven Shafizadeh, Sven Märdian, Gernot Steiner, Stephan Regenbogen

**Affiliations:** 1 Trauma and Orthopaedic Surgery, Cologne Merheim Medical Center, Witten/Herdecke University, Witten, DEU; 2 Center for Musculoskeletal Surgery, Charité - Berlin University of Medicine, Berlin, DEU; 3 Orthopaedics and Traumatology, Clinics of the City of Cologne, Cologne, DEU; 4 Orthopaedic Surgery, Trauma Surgery, and Sports Medicine, Witten/Herdecke University, Witten, DEU; 5 Trauma, Hand, and Reconstructive Surgery, Rostock University Medical Center, Rostock, DEU; 6 Orthopaedics and Trauma Surgery, Heidelberg University, BG Klinik Ludwigshafen, Ludwigshafen, DEU; 7 Traumatology and General Surgery, Murnau Accident Clinic, Murnau am Staffelsee, DEU

**Keywords:** acetabular fractures, femoral head fractures, high-energy trauma, hip injury, obturator hip dislocation, pipkin fractures, traumatic hip dislocation

## Abstract

Purpose

Traumatic hip dislocation into the obturator foramen is poorly understood due to the rarity of the injury. The purpose of this study was to analyze the mechanism of trauma, associated injuries, treatment, and complications in patients who sustained obturator hip dislocation and to evaluate long-term outcomes, including patient-reported outcome measures (PROMs).

Methods

Patient demographics, trauma mechanisms, concomitant injuries, and the treatment of all consecutive patients who sustained an obturator hip dislocation at three level-one trauma centers from 2009 to 2024 were analyzed. At follow-up, the incidence of avascular necrosis (AVN), post-traumatic osteoarthritis (PTOA), further complications, return to work and sports, and PROMs, including Tegner Activity Scale (TAS) and modified Harris Hip Score (mHHS), were recorded.

Results

A total of eight obturator hip dislocations were identified out of 201 traumatic hip dislocations. Associated knee injuries, particularly involving the posterior cruciate ligament (PCL), were observed in five of eight patients (62.5%). Computed tomography (CT) analysis revealed concomitant fractures of the femoral head (Pipkin Types I-IV) or acetabulum in six of eight cases (75%), with the femoral head involved in 50% of all cases. Five patients were followed up (mean 9.17 ± 4.79 years). All patients demonstrated a good or excellent mHHS (mean 85.44 ± 7.36), and the TAS slightly decreased compared to their pre-injury level (5.5 to 4.5). None of the patients had been diagnosed with PTOA or AVN or undergone total hip arthroplasty (THA).

Conclusion

Obturator hip dislocations most commonly result from a high-energy "dashboard injury" and are mainly associated with concomitant femoral head or anterior acetabular wall fractures. Long-term follow-up has shown only minor limitations in activities of daily living, sports, and return to work, with a low risk of femoral head AVN or PTOA.

## Introduction

Native hip dislocation is a potentially serious injury that most commonly results from high-energy trauma and is highly associated with complications such as avascular necrosis (AVN) of the femoral head, post-traumatic osteoarthritis (PTOA), nerve injury, and long-term limitations in activities of daily living [1,2]. While posterior dislocations have been studied more extensively, anteroinferior obturator dislocations remain poorly understood due to their rarity, accounting for only 4-7% of all hip dislocations [2-4]. The primary cause of posterior hip dislocation is thought to be a "dashboard injury," which involves impact with the knee while the hip is in flexion and adduction. Similarly, obturator hip dislocations are thought to result from high-energy trauma with the hip in abduction and external rotation. When a higher degree of flexion is reached, there is a potential for anteroinferior dislocation, and the femoral head may dislocate below the pubofemoral ligament, resulting in dislocation of the femoral head into the obturator foramen [5,6]. However, the exact mechanism underlying the trauma mechanism and the reasons why some hips dislocate posteriorly while others dislocate into the obturator foramen in response to similar trauma mechanisms remain poorly understood [2].

In posterior hip dislocations, there is evidence regarding characteristics, concomitant fractures, functional outcomes, and prognostic factors. However, the evidence for obturator dislocations is based on case reports and a few case series with small sample sizes [3-5].

Therefore, the purpose of this study was to analyze the mechanism of trauma, associated injuries, treatment, and complications in patients who sustained obturator hip dislocation. In addition, the study aimed to evaluate patient-reported outcome measures (PROMs) over a 15-year period with a minimum follow-up of 24 months. It was hypothesized that specific prognostic factors could be identified that would have an impact on the development of femoral head AVN or PTOA, PROMs, and return to sports.

## Materials and methods

After institutional review board approval, all consecutive patients aged ≥18 years who sustained a traumatic obturator hip dislocation at three level 1 trauma centers between 2009 and 2024 were included in the study and retrospectively analyzed. Exclusion criteria were incomplete medical records regarding the mechanism of trauma or treatment, or if their pelvic computed tomography (CT) scans were unavailable. Patient demographics, mechanism of trauma, concomitant injuries, time to reduction, treatment, and complications were assessed. CT scan analysis included the type of dislocation and concomitant acetabular or femoral head fractures. The latter were classified using the Pipkin classification system [7].

Follow-up and PROMs

Patients who met the inclusion criteria were enrolled prospectively after written consent was obtained. Patients with cognitive impairment or paraplegia were excluded from follow-up. The patient's history after hip dislocation was recorded, including re-dislocation, hip instability, AVN, PTOA, additional surgeries, nerve damage, and sexual dysfunction. Pre- and post-injury levels of sports activity were documented, as well as the patient's ability to resume previous levels of sports participation and return to work. In addition, pre- and post-injury activity levels were determined using the Tegner Activity Scale (TAS) [8]. The validated modified Harris Hip Score (mHHS) was used to assess activities of daily living, functional outcomes, and pain [9,10]. The results of the mHHS were classified as follows: <70 poor, 70-79 moderate, 80-89 good, and >90 excellent.

Statistical analysis

Statistical analysis was performed using Python (version 3.8.16; Python Software Foundation, Wilmington, DE, USA) within the Jupyter Notebook. Descriptive statistics, including means, frequencies, percentages, and ranges, were determined for continuous and categorical variables.

## Results

Patient demographics and trauma mechanism

A total of 201 consecutive native hip dislocations treated at our institutions over 15 years were analyzed. Following the inclusion and exclusion criteria, eight patients with obturator hip dislocation were finally included in the study, representing 3.9% of traumatic hip dislocations (Table 1).

**Table 1:** Demographics and trauma mechanism of patients with traumatic obturator hip dislocation (n=8) Values are expressed as numbers (N, %) or mean ± standard deviation (SD). BMI: Body mass index; ISS: Injury Severity Score

Variable	Value
Age at the time of injury	41.22 ± 22.73 years
Gender	
Male	6 (75%)
Female	2 (25%)
Side	
Right	2 (25%)
Left	6 (75%)
BMI	23.43 ± 4.36
Injury mechanism	
Motorcycle accident	1 (12.5%)
Suicidal collision with train	1 (12.5%)
Motor vehicle accident	2 (25%)
Skiing	1 (12.5%)
Fall from height	1 (12.5%)
Pedestrian collision with car	2 (25%)
Associated injuries	
Mono trauma	0
Multiple Injuries	1 (12.5%)
Polytrauma (ISS ≥16)	7 (87.5)
Duration of hospital stay (days)	11.93 ± 6.88

The mean age at the time of injury was 41.22 ± 22.73 years. All patients had sustained a high-energy trauma mechanism, with various types of traffic accidents and falls associated with polytrauma in almost all cases.

Concomitant fractures and associated injuries

**Figure 1:**
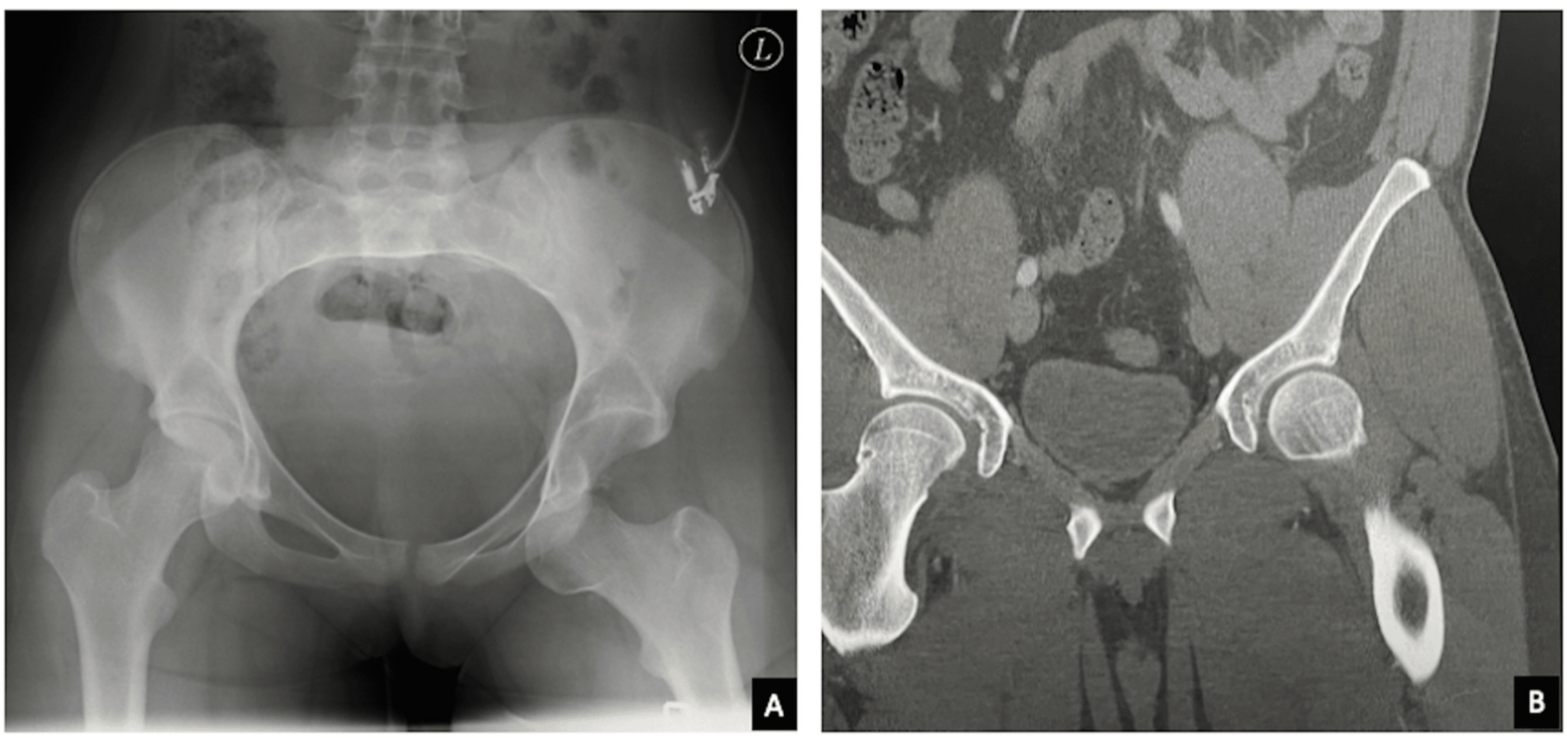
Radiographic image of a traumatic hip dislocation into the obturator foramen (A), and CT scan after closed reduction to detect concomitant acetabular or femoral head fractures, which were excluded in this case (B).

**Figure 2:**
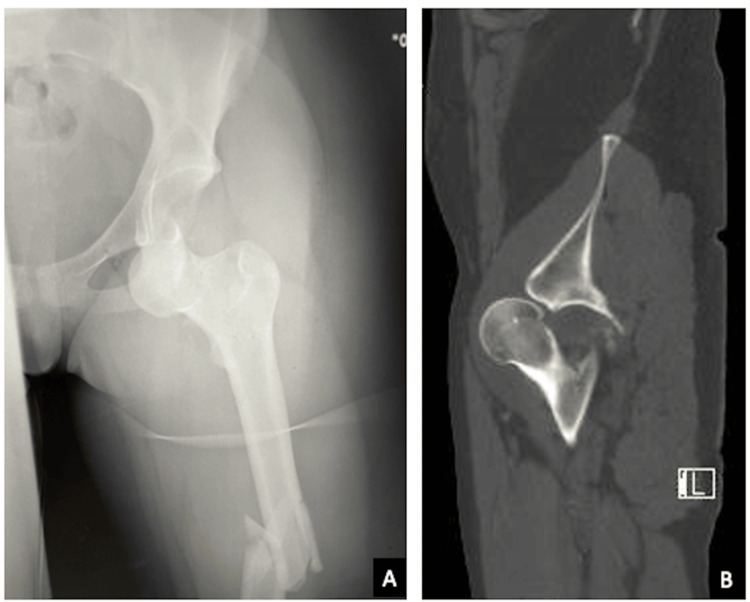
Radiograph of an obturator hip dislocation (A). The CT scan of the same patient reveals intra-articular fragments resulting from a concomitant fracture of the anterior wall of the acetabulum (B).

**Figure 3:**
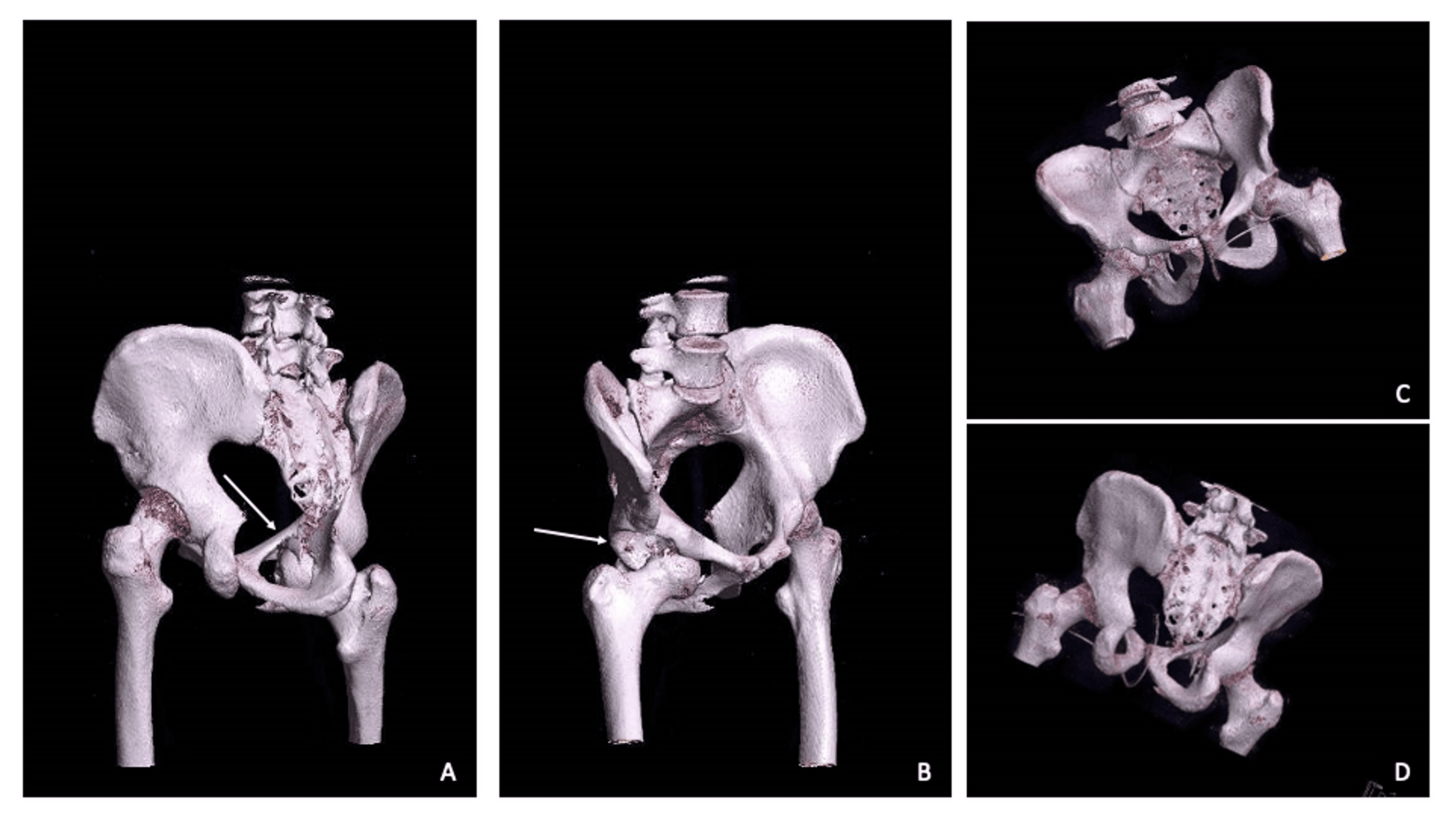
3D CT scans demonstrating a right obturator hip dislocation with concomitant fractures of the femoral head (arrow, A) and anterior acetabular wall (arrow, B), and after closed reduction (C, D)

CT scans after reduction were available in all cases and revealed concomitant fractures of the femoral head (Pipkin Types I-IV) or acetabulum in six of eight cases (75%), with the femoral head involved in 50% of all cases (Figures 1-3). In all cases, the associated acetabular fractures involved only the anterior wall. Associated knee injuries, particularly involving the posterior cruciate ligament (PCL), were observed in five of eight patients (62.5%) (Table 2).

**Table 2:** Concomitant fractures and associated injuries in patients with obturator hip dislocation (n=8) Values are expressed as numbers (N, %) or mean ± standard deviation (SD). PCL: Posterior cruciate ligament

Associated fractures	n (%)
Femoral head	4 (50)
Pipkin I	3 (37.5)
Pipkin II	0
Pipkin III	0
Pipkin IV	1 (12.5)
Isolated anterior acetabular wall	2 (25)
None (simple dislocation)	2 (25)
Ipsilateral knee injury	
Isolated PCL injury	2 (25)
Knee dislocation	2 (25)
Patella fracture	1 (12.5)
None	3 (37.5)
Primary sciatic nerve injury	
Yes	1 (12.5)
No	7 (87.5)

Primary treatment

**Figure 4:**
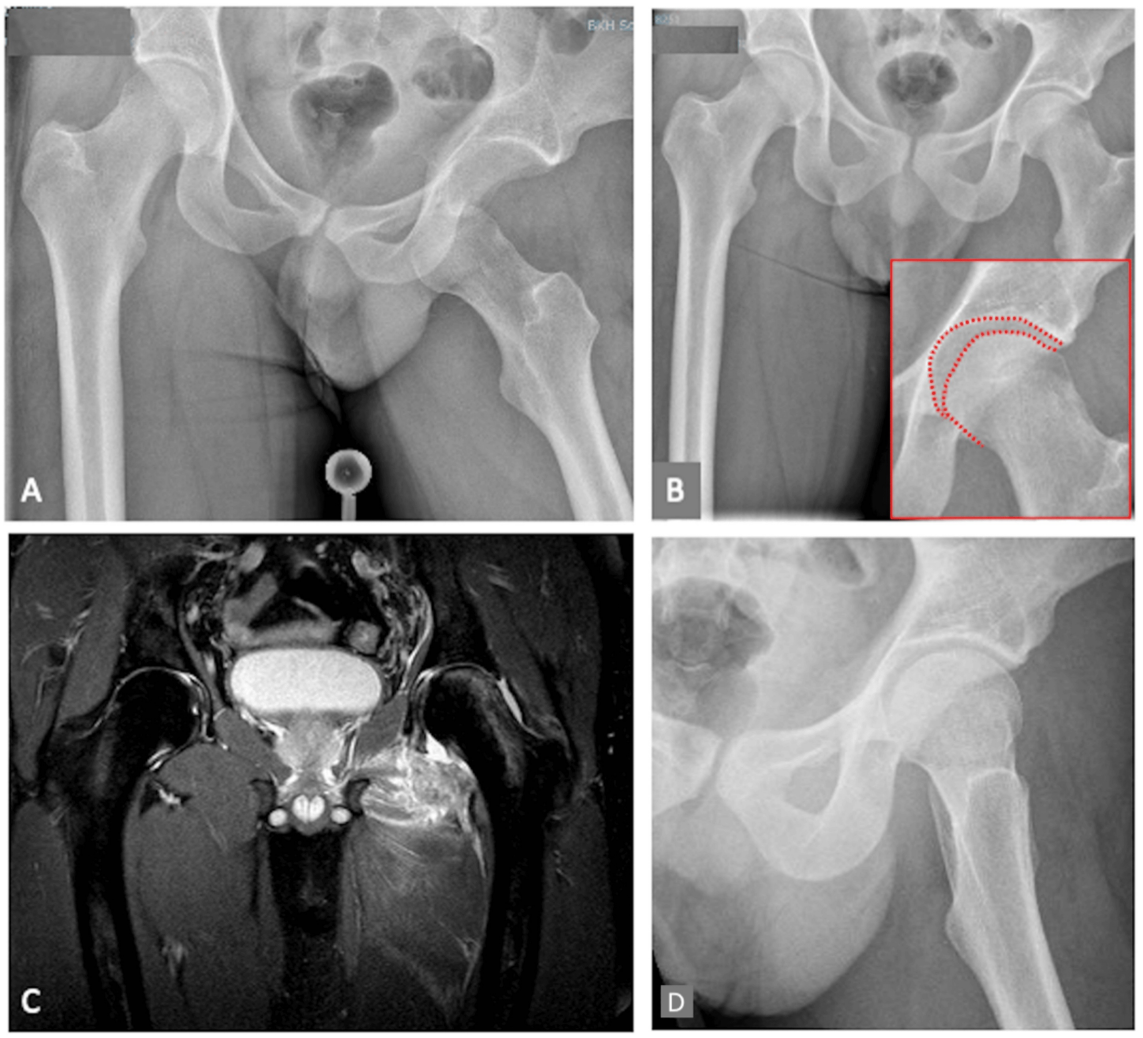
Radiographs of a traumatic obturator hip dislocation (A) and after closed reduction (B) demonstrating an incongruent joint line (red dotted line). The MRI revealed a caudal labral tear with free articular bodies (C). Therefore, arthroscopic labral refixation and removal of the free articular bodies was performed. The postoperative radiograph shows a congruent hip joint (D).

Closed hip reduction under sedation and relaxation was performed within six hours in all cases. In two cases, subsequent surgical procedures were performed with arthroscopic labral refixation with removal of remaining intra-articular fragments (Figure 4) and fixation of the Pipkin fragment, respectively. In the other cases, the concomitant fractures were not considered reducible because they were too small and remained outside the joint after closed reduction.

Follow-up and PROMs

Of the eight eligible patients, five (62.5%) were available for follow-up examination. The mean time to follow-up was 9.17 ± 4.79 years with a minimum follow-up time of >24 months. The mean age of the patients at the time of follow-up was 44.20 ± 12.78 years. None of the patients reported a re-dislocation of the hip or a residual subjective instability. No residual motor or sensory impairment of the sciatic or femoral nerves was observed. Posttraumatic sexual dysfunction was not reported. In addition, none of the patients had been diagnosed with PTOA or AVN, and none had undergone total hip arthroplasty (THA). All patients demonstrated good or excellent results in the mHHS. The mean TAS decreased slightly from 5.5 ± 2.62 before injury to 4.5 ± 2.50 at follow-up. Furthermore, all patients reported that they had no or only mild limitations in returning to their pre-injury level of sport. All patients were able to return to their previous work (Table 3).

**Table 3:** Clinical outcomes and patient-reported outcome measures (PROMs) (n=5) Values are expressed as numbers (N, %) or mean ± standard deviation (SD).

Variable	Value
Time to follow-up	9.17 ± 4.79 years
Age at follow-up	44.20 ± 12.78 years
Gender	
Male	4 (80%)
Female	1 (20%)
Modified Harris Hip Score	85.44 ± 7.36
Tegner Activity Scale	
Preinjury	5.5 ± 2.62
At follow-up	4.5± 2.50 years
Return to previous work	
Yes	5 (100%)
No	0
Limitations in sports activities (compared to preinjury)	
None	4 (80%)
Mild	1 (20%)
Moderate	0
Highly	0
Posttraumatic degenerations	
Osteoarthritis	0
Avascular necrosis	0

## Discussion

The results of this study provide insight into a better understanding of the underlying mechanisms, treatment, and clinical outcomes of traumatic obturator hip dislocations. At long-term follow-up, only minor limitations in activities of daily living, sports, and return to work were observed, with a low risk of PTOA or AVN of the femoral head.

Given the limited number of existing observational studies [4,6], the results of the present study support the hypothesis that high-energy trauma is the critical factor in obturator hip dislocation. While posterior hip dislocations are described as also being associated with lower-energy or sports-related mechanisms in up to 20% of cases [2,11], low-impact trauma has not been observed in patients with obturator hip dislocations.

All patients had sustained high-energy trauma, with a “dashboard injury” being the most likely mechanism, as associated knee injuries, particularly to the PCL, were found in nearly two-thirds of the patients. These findings are similar to the associated knee injuries in posterior hip dislocations [2,12,13]. Thus, the question of why some hips dislocate to the obturator foramen rather than posterior in “dashboard type” injury mechanisms is of interest. There may be an abduction or rotational force vector in addition to the axial energy that causes the hip to dislocate in an antero-inferior direction.

Previous studies have reported rates of concomitant fractures in anterior iliac or obturator hip dislocations with a wide range from 15% to 78% [3-5]. In the present study, associated fractures were observed in 75% of cases. The variability may be due to the variable use of cross-sectional imaging in previous studies, whereas the analysis of CT in this study allowed a precise evaluation in all cases. Consistent with previous findings by Wojahn et al. [4], most obturator dislocations were associated with simpler fracture patterns of the femoral head or anterior acetabular wall and were most commonly managed non-surgically.

The data of the present study demonstrate good or excellent functional outcomes, which are superior to those observed in posterior hip dislocations [2,14,15]. A possible explanation may be that anterior acetabular wall fractures may affect hip biomechanics less than posterior wall fractures in posterior dislocations. However, femoral head fractures were also observed in half of the cases, and these injuries are generally associated with a worse clinical outcome, as they are often associated with AVN [16-18]. However, this correlation did not hold true for the obturator hip dislocations with concomitant femoral head fractures examined in the present study, which is consistent with previous observations of low rates of AVN or PTOA in anterior hip dislocations [3,4]. Therefore, it can be hypothesized that the femoral head in the main load-bearing area of the joint is unlikely to be damaged by this type of dislocation.

There are strengths and limitations to this study. Previous evidence of obturator hip dislocation is based on case reports and a few case series due to the rarity of these injuries. Although our sample size is relatively small, it is one of the largest series with the longest follow-up period in the current literature. However, with respect to the small sample size, only descriptive data have been provided, as the sample size was too small to conduct significant subgroup analyses. The results are relevant to the management of this injury because surgeons may need to rely on more experience. As most of the patients had multiple injuries, associated injuries may have affected the functional outcome. However, this strengthens the results because most patients had good or excellent functional outcomes.

## Conclusions

In conclusion, this study provides insight into the epidemiology, injury characteristics, and clinical outcomes of traumatic hip dislocation into the obturator foramen. The injury mechanism is a high-energy impact and is commonly associated with knee injuries, specifically involving the PCL, suggesting a "dashboard" mechanism. Obturator hip dislocations are associated with femoral head or anterior acetabular wall fractures in the majority of cases. However, a relatively simple fracture pattern allows for nonoperative treatment in most cases. Furthermore, in long-term follow-up, only minor limitations in activities of daily living, sports, and return to work were observed, with a low risk of femoral head AVN or PTOA. In conclusion, the results of this study provide a better understanding of the underlying mechanisms, management, and clinical outcomes of traumatic obturator hip dislocations in one of the largest patient cohorts over 15 years at three level I trauma centers.
